# Genus Stachys—Phytochemistry, Traditional Medicinal Uses, and Future Perspectives

**DOI:** 10.3390/molecules29225345

**Published:** 2024-11-13

**Authors:** Stela Pashova, Diana Karcheva-Bahchevanska, Kalin Ivanov, Stanislava Ivanova

**Affiliations:** 1 Department of Pharmacognosy and Pharmaceutical Chemistry, Faculty of Pharmacy, Medical University of Plovdiv, 4002 Plovdiv, Bulgaria; stela.pashova@mu-plovdiv.bg (S.P.); diana.karcheva@mu-plovdiv.bg (D.K.-B.); kalin.ivanov@mu-plovdiv.bg (K.I.); 2 Research Institute, Medical University of Plovdiv, 4002 Plovdiv, Bulgaria

**Keywords:** *Stachys*, traditional medicine, phytochemistry, phytopharmaceuticals

## Abstract

The genus *Stachys* represents one of the most extensive genera within the subfamily *Lamioideae* and ranks as one of the largest genera in the *Lamiaceae* family. Many *Stachys* species are associated with a rich history in traditional medicine across various cultures, and their extracts and essential oils are rich in non-volatile and volatile compounds. Because of their complex profile of bioactive substances, *Stachys* members are considered to possess an extensive spectrum of therapeutic properties, including antioxidant, anti-inflammatory, analgesic, antibacterial, cytotoxic, and wound-healing effects, as well as benefits for memory enhancement, lipid profile regulation, blood glucose control, and weight management. Despite the wide distribution and chemical diversity of the genus, studies on its biological activities remain limited. The aim of this review is to summarize the most relevant data from studies on the bioactive compounds, traditional uses, and pharmacological properties of *Stachys* species found in databases such as Science Direct, PubMed, and Scopus. Specific keywords were used in the search strategy, including “bioactive compounds”, “Stachys”, “Lamiaceae”, “Stachys extract”, “Stachys essential oil”, “traditional uses”, “chemical composition”, “therapeutic potential”, “clinical trials”, “in vivo studies”, “in vitro studies”. The search strategy was performed according to the guidelines for Preferred Reporting Items for Systematic Reviews and Meta-Analyses. Data from 171 studies were included. The manuscript highlights the drug-discovery potential of *Stachys* species, emphasizing their potential applications in medicine, cosmetics, dietary supplements, and the food industry. Moreover, it provides important data that could assist Stachys research programs.

## 1. Introduction

The genus *Stachys* is one of the most expansive genera in the subfamily Lamioideae and ranks first among the biggest genera covered by the entire family Lamiaceae [1,2]. One of the most controversial issues related to the taxonomy of *Stachys* is its relationship with Betonica, which has been categorized by various authors as either a separate genus or subgenus [3,4].

About 300 species belong to the genus, including annual or perennial herbaceous species, and small shrubs. The genus is widely distributed in a diverse range of locations, demonstrating its cosmopolitan presence. However, it is notably absent in Australia and New Zealand [5,6]. North America, Central America, and Mexico show high species richness, while South America is characterized by moderate diversity [7,8,9]. Significant *Stachys* biodiversity can be observed in South and North Africa [6,10]. A great variety of *Stachys* species can be found in Europe and the countries of the former Soviet Union [11]. The Mediterranean region also features a wide range of *Stachys* species [6,12]. Turkey, the Middle East, and Iran exhibit notable levels of diversity, while China demonstrates a smaller spectrum of variety compared to the rest of the world [13,14,15].

Phylogenetic investigations reveal that the *Stachys* genealogy is divided into two clades [3,9]. The first clade, with its primary diversity concentrated in the eastern Mediterranean, has gradually migrated to encompass Western Asia, Western Europe, Macaronesia, and Sub-Saharan Africa [3,9]. In contrast, the second clade, comprising the Hawaiian mints, Suzukia, all species of *Stachys* indigenous to the New World, and certain Old World species, display signs of an Old World origin with subsequent movement to the Americas and Hawaii [1,16].

The etymology of the genus name derives from the Greek term “stachys (=στάχυς)”, referring to the shape of its inflorescence, which resembles a “spike of corn”. In the past, the word “Stachys” was mostly used for the plant *Stachys germanica* L., distinguished by its corncob-like inflorescence covered with pale hairs [17,18]. Trifarium, meaning “tomentose”, is the Latin name of the genus [17]. One of the common names of the genus is “woundwort”, originating from ancient traditions where certain plants of the genus were applied to heal wounds [19].

The genus *Stachys* includes mostly perennial or annual herbaceous plant species that are usually covered with hairs and are commonly characterized by a remarkable aroma [2,6]. The leaves of *Stachys* plants are usually ovate, occasionally lanceolate or heart-shaped, with serrated edges, and are typically attached to the stem by a petiole, although they may occasionally be sessile [2,6]. Their inflorescences are of a thyrsoid or racemoid structure [6]. The flowers are typically on pedicels, although they may sometimes be sessile [6]. The calyx is usually distinguished by morphological diversity, including variable lobes with a frequent occurrence of spines, along with a tube that typically has a spiked throat [6]. The color spectrum of the corolla ranges from bright to violet shades, and usually has two lips [2]. May to July is the time when they blossom and bear fruit, with June often being the peak flowering time [2].

The majority of the genus’ members live in upland meadows [2,12]. On the other hand, certain species favor riparian areas such as riverbanks or moister sites such as woodlands [2,20]. Some plant species exhibit adaptation to both soil characteristics and local climatic conditions, allowing them to thrive specifically in rock crevices, and are therefore considered chasmophytes [2]. Consequently, these plants often have a limited geographical distribution and are endemic to certain regions [2].

Numerous species within the genus *Stachys* are widely employed in conventional healthcare in various countries due to their diverse therapeutic benefits [17,18,21]. The *Stachys* plants are considered to possess an extensive spectrum of therapeutic advantages due to the phytochemical composition of these species [17]. The main groups of metabolites found in this genus’ species have been identified as flavonoids, iridoids, fatty acids, and phenolic acids [22]. Several important factors, including climate, soil properties, seasonal fluctuations, harvest timing, and geographical locations, have a significant impact on the chemical composition of different plant species [5].

The *Stachys* genus could be considered as a source of multiple bioactive substances with unique biological properties [23,24,25,26,27]. Plants of this genus have long been recognized as an essential part of the traditional healing practices in many nations worldwide, where they are used to cure and control a broad spectrum of medical ailments, including skin issues, neurological disorders, stomach upsets, and inflammatory conditions [5,23,28,29,30,31,32]. In folk medicine, *Stachys* species are also used for their psychotropic and anxiolytic effects [30,33]. Different *Stachys* species have been used in the preparation of tea and the treatment of respiratory diseases [34,35]. Recent investigations have reported significant cytotoxic and antioxidant potential of several extracts prepared from different *Stachys* species including the following: *S. aegyptica*, *S. parviflora*, *S. affinis*, *S. lavandulifolia*, and *S. cretica* [36,37,38,39,40]. In addition to their medicinal applications, some plants have been served as spices in a variety of dishes and drinks [18].

According to recent studies and traditional medical knowledge, *Stachys* species have the potential to be incorporated into innovative herbal products, dietary supplements, and functional foods [34,41]. Nowadays, there is a growing demand for natural products in conventional healthcare, the food industry, cosmetics, and ornamental horticulture. This trend has led to the expansion of cultivation and research of multiple plant species including *Stachys* species [19,34].

This study aims to provide an in-depth assessment of the medicinal applications of the genus *Stachys* L., highlighting its therapeutic potential and future perspectives for use in medicine and the pharmaceutical industry, which will support the study, evaluation, and extraction of specific molecules from plants belonging to the genera.

This is the first study to compare and explore the phytochemical profile of 45 Stachys species. Moreover, the article provides data about in vivo, in vitro, and clinical trials based on these species. The article provides valuable data about the species and serves as a good starting point for researchers who would like to explore the therapeutic potential of Stachys.

## 2. Results and Discussion

### 2.1. Stachys Species—Phytochemistry

The *Stachys* genus includes a wide variety of plant species distinguished by their complex chemical profiles, including both volatile and non-volatile compounds. The chemical diversity of the *Stachys* genus members highlights their potential medicinal uses. The various types of non-volatile compounds identified in plants of the genus are presented in Table 1.

**Figure 1:**
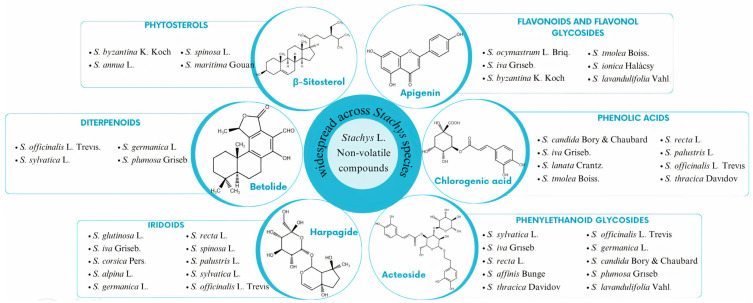
Non-volatile compounds widespread across *Stachys* species.

**Figure 2:**
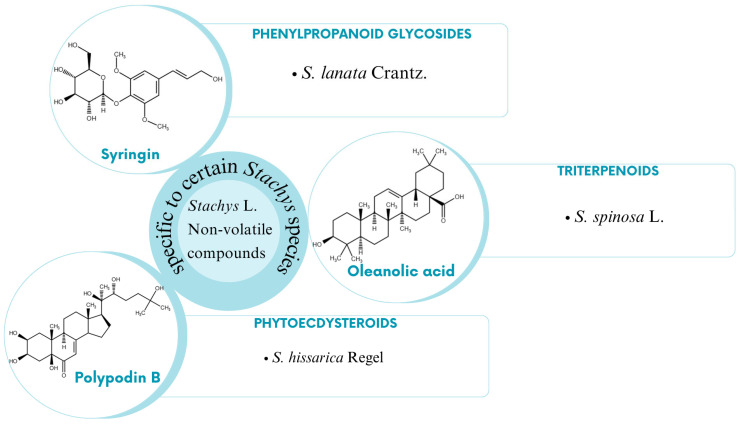
Non-volatile compounds specific to certain *Stachys* species.

A broad spectrum of non-volatile chemical compounds has been identified in different species of the genus (Figure 1 and Figure 2). These are divided into several categories, including flavonoids, phenolic acids, phenylethanoid glycosides, iridoids, diterpenoids, triterpenoids, phytosterols, and phytoecdysteroids.

*Stachys* is a genus characterized by an abundance of flavonoids. Most of them are isolated from the aerial parts. Noteworthy is the presence of apigenin, which has been detected in a huge number of species [42,43,44,45,46,47,48,49,50,51,52,53]. Apigenin is associated with diverse effects. It has a positive impact on diabetes, cancer, depression, insomnia, inflammation, and other diseases [87]. Many representatives of the *Stachys* genus are distinguished by the content of specific flavonoids [42,43,45,49,51,52,53,58]. For example, *S. lanata*, contains anisopholin A, a compound with antimycobacterial activity [42,88]. *S. ionica* is notable for possessing casticin, known for its anti-tumor, anti-inflammatory, analgesic, and neuroprotective properties, as well as salvigenin, which inhibits cancer cell growth and lowers lipid levels [52,89,90,91,92,93]. *S. lavandulifolia* is distinguished by velutin, which inhibits melanogenesis and cell differentiation, and viscosine, which exhibits anxiolytic, anticonvulsant, and hepatoprotective properties [53,94,95,96,97].

Furthermore, chlorogenic acid appears to be the major phenolic acid [28,42,47,49,50,55,62,63,69,70]. It could be isolated not only from the aerial parts but also from the roots [42,62,63]. One representative that contains chlorogenic acid in their roots are the Bulgarian plant species of the genus *Stachys*, as well as *S. thracica*, and *S. lanata*, cultivated in Japan [42,62,63]. Chlorogenic acid occurs naturally in green coffee and tea. It exhibits a wide range of activities—antioxidant, antibacterial, antiviral, and anti-inflammatory. In addition, chlorogenic acid has been associated with positive effects on lipid and glucose metabolism [98].

Phenylethanoid glycosides are phenolic compounds that are present in a wide range of medicinal plants, especially in the families Lamiaceae, Plantaginaceae, Scrophulariaceae, and Orobanchaceae. This group of compounds has numerous beneficial properties, such as those antiviral, antibacterial, anti-inflammatory, antidiabetic, and neuroprotective characteristics. Although more than 570 phenylethanoid glycosides have been identified, only a few have been thoroughly investigated, and their limited bioavailability restricts their value as medicinal agents [99]. Regarding the phenylethanoid glycosides, acteoside is reported in the majority of *Stachys* species [28,47,55,62,63,64,71,72,74,83]. In contrast, there are constituents belonging to this group that are mentioned only in certain species. Betonyosides are found only in *S. officinalis* and *S. recta*, while stachysosides are typical for *S. sieboldii* [28,72,76]. Furthermore, parvifloroside A and parvifloroside B are common in *S. parviflora*, while darendoside B and rhodioloside are characteristic of *S. lanata* [42,75].

Iridoids are among the main chemical substances occurring in the genus *Stachys* [28,47,52,68,71,73,77,78,79,80]. Harpagide and acetylharpagide are the two most frequently detected compounds of this class [28,47,68,71,77,78,79]. Harpagide, which is extensively distributed in the Lamiaceae family, serves as an important chemotaxonomic indicator. It occurs primarily in genera such as *Stachys* L. and *Ajuga* L. This compound demonstrates a broad spectrum of pharmacological activities, including anticancer, antibacterial, anti-inflammatory, antidiabetic, spasmolytic, hepatoprotective, and neuroprotective properties [100]. Meanwhile, acetylharpagide is recognized for its antipyretic, cardiotonic, and anti-fungal effects [101]. In addition, aucubin, ajugoside, and catalpol are other commonly observed constituents in the genus that exhibit diverse biological effects [79]. With characteristics such as anti-aging, anti-fibrotic, anticancer, neuroprotective, and hepatoprotective effects, aucubin is a very promising compound [102]. The pharmacological potential of ajugoside in *Stachys* species is highlighted by its strong antioxidant, hypoglycemic, cardioprotective, anti-inflammatory, and anticancer activities [103]. Similarly, catalpol exhibits almost the same broad spectrum of biological activities [104]. *S. ocymastrum* is the only member of the genus that contains hydroxyipolamiide, known for its antioxidant and antidiabetic characteristics, ipolamiide, noted for its antimicrobial activity, ipolamiidoside, recognized for its antiviral properties, and lamiide for its antioxidant activity, all found in the leaf extract of the plant [80,105,106,107,108].

*S. officinalis* is unique among *Stachys* species for its high content of diterpenoids in its roots [81].

*S. hissarica* is a representative of the genus, characterized by the presence of phytoecdysteroids, including deoxyecdysone, integristeron A, and polypodin B [84]. Phytoecdysteroids are known for their diverse biological activities, such as antioxidant, anabolic, adaptogenic, anti-inflammatory, and anticancer properties [109]. Among phytosterols, β-sitosterol and stigmasterol are components isolated from several species. Phytosterols possess considerable therapeutic potential due to their capacity to regulate lipid metabolism and influence cell development and proliferation [110]. Within the genus *Stachys*, the halophytic plant *S. maritima*, growing on the Bulgarian Black Sea coast; the Greek *S. spinosa*; and *S. byzantina*, found in Iran, are recognized as sources of phytosterols [39,61,86].

All these properties of the compounds identified by the representatives of the genus *Stachys* highlight its significant medicinal value. The extensive range of health advantages provided by these *Stachys* species is evidenced by the various biological effects of the chemical groups investigated. This thorough pharmacological profile suggests that many *Stachys* members could be utilized in functional foods, dietary supplements, and cosmetics after more in-depth investigations.

Many members of the genus are also sources of essential oils (EOs) [23,29,111,112,113,114,115,116,117,118,119]. Despite belonging to the same genus, there are notable variations between EOs obtained from different species. The chemical composition of the EOs may differ because the species grow in various locations all over the world, under completely different climatic conditions. In Table 2, differences in the chemical composition of the EOs of *Stachys* species could be observed.

Turkey is one of the countries rich in various *Stachys* species [29,111,112]. Among the species investigated, it is notable that they possess high levels of β-caryophyllene and germacrene D. The species with the highest amount of β-caryopyllene was *S. viticina* (62.3%), followed by *S. cretica* subsp. *smyrnaea* (51.0%) [29,112]. In contrast, *S. recta* has a relatively lower β-caryophyllene content of 3.7% [111].

In general, EOs isolated from *Cinnamomum tamala* (25.3%) and *Cannabis sativa* (between 3.8% and 37.5%) are regarded as rich sources of the natural bicyclic sesquiterpene β-caryophyllene [120]. However, it seems that this compound could be isolated in high concentrations from plant species belonging to the *Stachys* genera, including *S. balansae*, *S. cretica* subsp. *smyrnaea*, *S. cretica* subsp. *bulgarica*, *S. viticina*, *S. obliqua*, *S. sericantha*, *S. huetii*, *S. tmolea*, *S. germanica* subsp. *bithynica*, *S. germanica* subsp. *heldreichii*, *S. iva*, *S. scardica*, and *S. officinalis* [29,111,112,113,114,115].

The cannabinoid receptor type 2 agonist β-caryophyllene is currently attracting considerable interest. The compound is largely considered safe, and at the same time, has a significant potential to be involved in the prevention and management of different neuroinflammatory and neurodegenerative pathologies and other conditions [121]. Numerous studies have reported that this cannabinoid is able to inhibit the activity of nitric oxide synthase and to enhance the activity of the antioxidant enzymes by affecting lipid peroxidation and glutathione levels [122,123]. β-caryophyllene has been reported to suppress the expression of Il-6 and IL-1β, which are related to the inflammatory process [120].

In the past decade, the compound has been studied for its potential to be included in the treatment of obesity and overweight. Supplemental β-caryophyllene intake has been found to have beneficial effects in obese individuals, including weight loss and reduction in body fat percentage [124,125].

Germacrene D also plays a crucial role in the phytochemistry of plant species. In addition to exhibiting insecticidal action against mosquitoes and repellent activity against aphids and ticks, this substance could also repel herbivores [126]. Furthermore, germacrene D could be considered a plant pheromone that stimulates interaction between various species [127]. Initially, germacrene D was derived from *Pseudotsuga japonica* EO [128]. High levels of germacrene D were found in the EOs of species belonging to *Beilschmiedia*, *Cedrela*, *Croton*, *Eugenia*, *Ocotea*, and *Piper* [129]. In general, germacrene D was present in considerable amounts in almost all the examined EOs of *Stachys* plants grown in Turkey. It is unusual for its content to be much lower. *S. obliqua* exhibited the greatest concentration of germacrene D among Turkish representatives, accounting for 45.3% [29]. S. bayburtensis and *S. sericantha* had nearly identical values, of 33.4% and 32.4%, respectively [29]. Germacrene D was not among the principal volatile compounds found in *S. balansae* and *S. recta* L. [111]. It is a component of *S. viticina* EO, but not part of the major compounds, accounting for only 2.9% of the total EO [29].

Croatia is another country where *Stachys* species are frequently discovered [113]. These species vary in their chemical profiles, even if they are distributed in the same region. Similarly to Turkey, germacrene D presents above the main volatile components, but at a lower volume. The two species richest in germacrene D are *S. salvifolia* (22.3%) and *S. officinalis* (20.1%) [113]. The concentrations of germacrene D were lowest in *S. recta* subsp. recta and *S. palustris*, with 3.4% and 3.5%, respectively [113].

Several *Stachys* species exist in the Former Yugoslavian Republic of Macedonia [114]. Table 2 provides information on the chemical composition of EOs obtained from two species distributed in this country: *S. germanica* subsp. *heldreichii* and *S. iva* [114]. Their main constituents are more than those identified in the EOs of *Stachys* species in Turkey and Croatia. These species contain a large amount of volatile substances, with (E)-nerolidol being dominant in the chemical profile of *S. germanica* subsp. *heldreichii* with 13.5% [114]. Nerolidol, or peruviol, is a naturally occurring sesquiterpene alcohol that is florally scented and is found in the EOs of many plants. It exists in two geometric forms: trans and cis. The predominant form found in EOs of *Stachys* species is trans. Nerolidol is a common ingredient in cosmetics as well as in the food industry as a flavor enhancer. The source with the highest concentration of (E)-Nerolidol is *Piper claussenianum* (81.4%), followed by *Zanthoxylum hyemale* (51.0%) [130]. *Stachys* species are not distinguished by their high percentage of nerolidol. However, the presence of this substance in the composition of EOs leads to effects that are typical of nerolidol, including antibacterial, antioxidant, repellent, and anticancer effects, as well as enhanced skin penetration [130]. In contrast, the most common component in *S. iva* is (Z)-Nuciferyl isobutyrate, accounting for 14.0% of the total EO. This compound is also among the main constituents in *S. germanica* ssp. *heldreichii* EO, but at a lower concentration (5.5%). In general, nuciferyl esters have been identified as secondary metabolites mostly in the Asteraceae family, and, more precisely, in the genera *Artemisia* and *Pulicaria*. So far, there is no evidence of the presence of nuciferyl esters in any other *Stachys* species or other Lamiaceae members [114].

Another Balkan area where *Stachys* species may be identified is Serbia [114,115]. In addition to Croatia, *S. officinalis* can also be found in Serbia [113,115]. There are similarities in their chemical profiles. Both EOs contain germacrene D and β-caryophyllene as major compounds, suggesting some regional consistency. The variations are related to the concentrations of specific volatile compounds. The amount of α-humulene in the Serbian EO is greater than that of the Croatian EO [113,115]. However, the Croatian sample showed a slightly higher level of (E)-caryophyllene [113]. Other *Stachys* species identified in Serbia are *S. plumosa* and *S. scardica*, each of which exhibits an individual chemical profile consisting of major volatile compounds along with varying levels of other constituents [114,115]. The main volatile ingredient in *S. plumosa* is ar-abietatriene (45.5%), while *S. scardica* possesses γ-muurolene (13.1%) [114,115]. Caryophyllene oxide is a typical constituent in all three Serbian species, but in different amounts [114,115].

*Stachys* species occurring in Greece have an array of volatile compounds [23,116]. Each species possesses a unique set of volatile components. *S. sprunieri* exhibits a high content of (+)-limoene (17.3%), whereas *S. ionica* contains significant quantities of (E)-neolidol (14.9%) and α-cadinol (13.1%) [116]. *S. spinosa* is the Greek *Stachys* species, remarkable for its content of significant amounts of carvacrol (27.9%), although all other components are substantially lower in level, each accounting for less than 5% of the total EO [23]. The main sources of carvacrol are considered to be the genera *Origanum*, *Thymus*, *Thymbra*, *Satureja*, and *Coridothymus* of the Lamiaceae family, all included in the term Oregano. Oregano has gained popularity since ancient times. Discovering its antibacterial effects, Hippocrates applied it to treat respiratory and gastrointestinal conditions. Moreover, Dioscorides prescribed the consumption of a mixture of oregano tea and wine as a cure for snake bites. Nowadays, carvacrol is still widely used in a variety of products due to its broad spectrum of effects, such as antibacterial, anticancer, analgesic, insecticidal, and hepatoprotective [131].

Several *Stachys* species are also found in Iran [117,118]. Among them is *S. balansae* [117]. It has also been discovered in Turkey [111]. Significant variations were observed in the chemical profiles of the two species [111,117]. Although both of them contain α-pinene and β-pinene as main compounds, their proportions differ considerably [111,117]. Germacrene D is the predominant component in the Iranian species, while β-caryophyllene is the most abundant compound in the Turkish *S. balansae* [111,117]. *S. schtschegleevii* and *S. byzantina* are the other representatives of the genus *Stachys* that occur in Iran [117,118]. *S. byzantina* stands out for its high content of hexahydrofarnesyl acetone, whereas *S. schtschegleevii* possesses relatively greater levels of germacrene D [117,118].

Although there is almost no data on the chemical composition of the essential oils of *Stachys* species growing in Bulgaria, a study reveals key differences when compared to other EOs [119]. *S. germanica* shows the presence of more than 50% camphor and could therefore be considered as an alternative source [119]. Camphor is a bicyclic monoterpene, which could be identified in many EOs [132]. Numerous applications of camphor have been known for long periods. It is extensively used as an aromatic component in cosmetics, a flavoring ingredient, a preservative, and a repellent. Additionally, camphor exhibits antimicrobial, antitussive, and analgesic effects. It is a common constituent in topical analgesics, but is toxic if administered internally [133].

Similarly, a lack of data on the EO derived from *Stachys* species is available in Lebanon [23]. A studied member of the genus is *S. nivea* [23]. Its EO is defined by low concentrations of volatile compounds, with spathulenol at the highest concentration of 6.7% [23].

**Figure 3:**
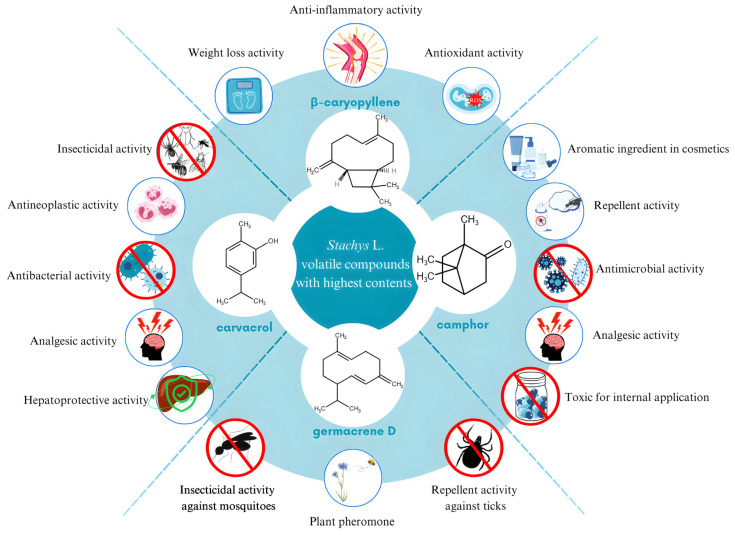
Major volatile compounds and biological activities of *Stachys* species.

The wide range of volatile and non-volatile compounds found in *Stachys* species highlights their chemical diversity, emphasizing the necessity for further investigation of their biological activities and potential applications (Figure 3). The existence of distinct compounds in each species may contribute to their therapeutic characteristics, requiring additional research on their pharmacological activity.

### 2.2. Traditional Medicinal Uses of the Genus Stachys

In ancient cultures, plants played a significant role in healing practices. Their importance in therapy remains essential even today [134]. Developing nations have integrated herbal remedies as substitute therapies for many medical conditions [135].

For millennia, numerous species within the genus *Stachys* have been employed worldwide in ethnomedicine. Many scientific investigations have confirmed their extensive range of traditional therapeutic applications [47]. Multiple species belonging to this genus have become popular in traditional healthcare, with different preparations are used for the management of a range of diseases [47].

According to the data presented in Table 3, *Stachys* species are distributed across different regions worldwide and are used for the treatment of various ailments.

A wide variety of *Stachys* species can be found in Iran and Turkey [5,32,34,73,134,142,144,146,147,148,154,156,157]. In Iran, there is a strong correlation between the local flora and traditional healing methods, as demonstrated by the abundance of *Stachys* species identified there [32,73,144,146,147,148,154,156]. A total of 34 species of the genus *Stachys* are recognized in different geographical locations in Iran, with 13 of them being indigenous to the country [154]. Turkey also stands out as a center of diversity for *Stachys* [5,34,134,142,157]. The broad range of *Stachys* species demonstrates the value of herbal medicines in local healthcare systems.

Moreover, Asian regions, Brazil, Poland, Scandinavia, and the Mediterranean region, which includes Italy and Greece, contribute to the global distribution of *Stachys* species, demonstrating the broad application of these plants across a variety of climates and countries [32,41,47,71,135,136,137,138,139,140,141,145,149,150,151,155,158]. The Mediterranean region is characterized by remarkable plant biodiversity, both because environmental conditions promote the diversification of new species and because new species easily adapt to this environment [159,160].

Table 3 shows the variety of traditional therapeutic uses of *Stachys* species in diverse regions, indicating their potential as natural sources of medicines for a broad spectrum of conditions such as gastrointestinal problems, pain, respiratory disorders, and colds, in addition to their antioxidant, antibacterial, and sedative properties. Certain *Stachys* species have traditionally been used to treat cold symptoms across various geographical areas. These species, all endemic to Turkey, are *S. annua* subsp. *annua* var. *lycaonica*, *S. iberica* subsp. *georgica*, *S. kurdica* var. *kurdica*, and *S. obliqua* [34,134,142]. They are utilized for preparing decoctions, infusions, and teas. *S. annua* subsp. *annua* var. *lycaonica* is also known for its impact on febrile conditions, which complements its spectrum of action [142]. *S. iberica* subsp. *georgica* and *S. kurdica* var. *kurdica* are also notable for their ability to reduce body temperature [134,142]. *S. obliqua* also has similarities with them. Additionally, *S. obliqua* has been applied to manage gastric discomfort [34]. *S. iva*, indigenous to Greece, has demonstrated effectiveness in preventing colds during the winter [47].

Some *Stachys* species possess characteristics that promote skin health and wound healing. They are applied topically to treat numerous dermatological disorders, including skin inflammation, infected wounds, and promoting wound-healing. Plants of the genus *Stachys* with such properties are *S. byzantina*, *S. germanica*, *S. officinalis*, *S. palustris*, *S. mucronata*, and *S. sylvatica* [32,39,144,146,149,150]. *S. byzantina*, which typically occurs in Iran, is extensively appreciated for its ability to heal infected wounds [144]. Another representative found in Iran is *S. germanca*, in infusion form, which is especially beneficial for treating animal dermatological issues [32,146]. *S. officinalis*, utilized in Italy, has a similar application in veterinary medicine [150]. *S. palustris* is recognized not only for its wound-healing effect but also for its disinfectant characteristics and therapeutic effects on spasms [39,151]. The action of *S. sylvatica* is similar [39,143,156,157]. In Greece, people traditionally used a decoction of *S. mucronata* to clean wounds and ulcers and then cover them with a poultice made of fresh leaves to promote cicatrization and healing [149]. Furthermore, the decoction was applied topically to relieve rheumatic and neuralgic discomfort [149]. An infusion of roots provides purgative properties, while an infusion of the leaves promotes antidiarrheal effects [149].

*Stachys* representatives are also well-known for their analgesic effect, providing pain relief for a wide range of diseases. Examples of these plants include *S. inflata*, *S. lavadulifolia*, *S. pilifera*, *S. annua*, *S. officinalis*, *S. parviflora*, *S. recta* L., and *S. turcomanica* [32,34,73,146,147,151,152,153,154,155]. *S. lavadulifolia*, found in Iran and Turkey, exhibits diverse therapeutic applications in different regions [5,73,142,147]. In Turkey, an extract of this plant is served because of its effectiveness against anxiety disorders [5,142]. In Iran, it is utilized as a decoction to relieve head and stomach discomfort and kidney stones, while the extract has painkilling and anti-inflammatory effects [73,147]. In Iranian folk medicine, decoctions of both *S. pilifera* and *S. turcomanica* are employed against toothaches [146,147,154]. In the Mediterranean region, *S. annua* and *S. recta* are well-known for their topical application in alleviating headaches [32,155]. The leaves of *S. officinalis* were utilized for the same purpose [34,151]. In contrast, *S. parviflora* is used for treating arthralgia, cramps, and joint discomfort [152,153].

Therapies for respiratory disorders such as bronchitis, asthma, and cough are regularly helped by *Stachys* species. They assist in clearing the airways, suppress coughing, and facilitate breathing. This highlights the value of *Stachys* plants in conventional medicine as effective solutions for respiratory conditions. *S. obliqua* and *S. pilifera* are both associated with coughing, as the former has an antitussive activity and the latter possesses an expectorant effect [34,147,154]. On the other hand, *S. inflata* is associated with the control of asthmatic conditions [148]. The species *S. tibetica* is useful in the treatment of respiratory conditions due to its well-known bronchodilator properties [158]. Additionally, it possesses antitussive and antipyretic effects [158]. Moreover, it is employed to deal with diverse mental problems [158].

There are *Stachys* species that are associated with the function of the cardiac system. Beyond its broad therapeutic spectrum, which includes rheumatic and inflammatory issues, *S. inflata* is particularly useful in the treatment of hypertension and cardiac ailments [147,148]. Conversely, *S. balansae* is most commonly associated with hypotonic states and cardiac neuroses [143]. Similarly, *S. lanata* and *S. sylvatica* exhibit hypotonic effects, suggesting potential roles in cardiovascular health and blood pressure regulation [143,157].

Some members of the genus *Stachys* are specific to certain areas and possess unique qualities, which has led to their widespread usage in traditional medicine. In China, *S. affinis* has been employed for serious diseases, including tuberculosis, pneumonia, and urinary tract infections [71,136,137,138,139,140]. On the other hand, *S. officinalis*, found in Serbia, Montenegro, and Egypt, has been used to treat menopausal symptoms and has also been shown to help with smoking cessation [34]. Another representative is *S. sylvatica*, which affects the contractility of the uterus [143]. Its activity is comparable to that of ergotoxine, and this has allowed *S. sylvatica* to be suggested for use in obstetric–gynecological therapy after childbirth, as well as in cases of hemorrhage [143].

Despite geographical diversity, certain traditional uses are similar across regions. For example, many *Stachys* species are used to treat gastrointestinal problems, respiratory conditions, and colds, demonstrating shared traditional knowledge about the medicinal characteristics of these plants.

Moreover, some species of the genus offer nutritional value because of the importance of their edible tubers as a carbohydrate source [137]. An example is *S. affinis*, also recognized as Chinese artichoke [71]. The tubers of this plant species can be consumed in a variety of ways, including fresh, cooked, or pickled [71]. The tubers are renowned for their high Fe2+ content, making them a good nutritional choice for people who suffer from anemia [71]. *S. palustris* L., which occurs in the Scandinavian area, is another example that is safe for consumption and has rich nutritional value in its dried tubers [151].

Further research is needed to discover safer remedies for various conditions, and ethnomedicine could be a base for such investigations.

### 2.3. Therapeutic Potential of the Genus Stachys

Although *Stachys* species have been used for a long time, not many clinical trials have been conducted to evaluate their therapeutic benefits or potential negative effects. Further double-blind randomized trials are required to fully assess their efficacy and safety profile.

Table 4 summarizes the main benefits of several *Stachys* species that have been proven by clinical trials.

The data presented in the table above clearly reveal that the number of clinical studies is relatively limited, with only one species of the genus *Stachys*, *S. lavandulifolia*, having been studied to date.

People are constantly exposed to environmental pollutants, stress, and various diseases in their daily lives. These conditions lead to oxidative stress, which damages vital biomolecules. This oxidative stress, caused by an imbalance in free radical generation and scavenging, has been linked to cancer, autoimmune disorders, inflammation, and the aging process. The administration of the extract of *S. lavandulifolia* allows for a considerable reduction in oxidative stress [161].

Additionally, *S. lavandulifolia* affects conditions prevalent among women, such as menstrual discomfort and polycystic ovary syndrome (PCOS). Herbal medicines are becoming popular for the treatment of PCOS because of their minimal negative effects. Side effects of *S. lavandulifolia* are limited to stomach cramps and bloating, which are less unpleasant than the nausea and vomiting associated with medroxyprogesterone acetate (MPA). Other side effects of MPA, including weight gain and melasma, further restrict its use. The aerial parts of *S. lavandulifolia* are a rich source of apigenin, a flavonoid with estrogenic properties. However, further research is needed to clarify its influence on PCOS. Moreover, *S. lavandulifolia* contains other flavonoids that have been observed to play a crucial role in the regulation of the hypothalamus–pituitary–adrenal axis. Given the less pronounced side effects of *S. lavandulifolia* compared to MPA, the plant could be considered as an alternative treatment for the clinical symptoms of PCOS [162]. The influence of *S. sylvatica* on PCOS has also been observed in an in vivo study. Additionally, the study demonstrated that the extract of *S. sylvatica* has a beneficial effect on the control of obesity, which is one of the symptoms of this condition [156].

Dysmenorrhea is another prevalent gynecological problem among women. While NSAIDs are commonly prescribed for the treatment of this condition, they may induce gastrointestinal side effects, whereas *S. lavandulifolia* has demonstrated gastroprotective properties. Thymol, found in *S. lavandulifolia*, has been documented in the literature for its potential antispasmodic effects. *S. lavandulifolia* has been reported to alleviate menstrual discomfort and can be taken in combination with NSAIDs to minimize the frequency of their side effects. The herbal preparation could be used as a safer alternative to NSAIDs. However, longer follow-up studies are needed to evaluate its efficacy and safety profile [163].

*S. lavandulifolia* is effective not only in minimizing menstrual discomfort but also in reducing migraine pain. Migraine, the most common type of headache, leads to challenges due to its impact on quality of life and work productivity. As a result, the use of safe medications for migraine therapy is important. *S. lavandulifolia* represents such a treatment due to its antioxidant and anti-inflammatory properties [164].

Although few clinical trials are available, a larger amount of data is accessible from in vivo studies on various species within the *Stachys* genus (Table 5).

An in vivo study demonstrated that *S. lavandulifolia* essential oil exhibited considerable antinociceptive and anti-inflammatory properties. This EO is a rich source of α-bisabolol, a monocyclic sesquiterpene alcohol often used in dermatological products. Its ability to inhibit TNF-α and IL-1β highlights its potential as an anti-inflammatory agent. Despite these promising results, further investigations are necessary to determine the specific mechanisms of action [165]. *S. glutinosa* is also known for its similar action, attributed to xanthomicrol, a major compound in the chemical composition of the dichloromethane extract of the plant. Xanthomicrol reduces pain by binding to μ and δ opioid receptors [57].

Another member of the genus *Stachys* that has been investigated is *S. sieboldii*, also known as *S. affinis*. Commonly referred to as Chinese artichoke or chorogi, this plant is widely consumed as food in Japan. Apart from its use in cuisine, it has also been used in traditional medicine, prompting research inquiries. Studies have focused on its potential effects on memory and blood glucose levels, both of which are connected to optimal brain function [139,166]. Further research discovered that the anti-nephritic activity of *S. sieboldii* is attributed to acteoside, one of its principal components, derived from the aerial parts of the plant [167]. Moreover, the impact of *S. sieboldii* on metabolism has been examined, notably in terms of obesity and dyslipidemia. It has been suggested that the powder from the roots of the plant may have anti-adipogenic and lipid-reducing effects by boosting lipid metabolism [168]. The extract of *S. pilifera* also exhibits a positive effect on liver function, which is probably due to its antioxidant activity [169,170].

The rapid wound-healing observed with treatment by *S. hissarica* is also notable. Presumably, the synergistic action of its main components, including ecdysteroids, contributes to this effect. *S. hissarica* extract produced remarkable results even in animals with alloxan-induced diabetes, where wound-healing is typically a much more prolonged process [84].

In vitro studies, shown in Table 6, have demonstrated that various representatives of the genus *Stachys* can exhibit a range of effects.

For instance, *S. aegyptiaca* exhibits cytotoxic effects attributed to stachysolone diacetate, while *S. cretica* has demonstrated antioxidant properties and an anti-Alzheimer’s effect, possibly through the inhibition of acetylcholinesterase [49,171]. Additionally, *S. cretica* shows an antidiabetic effect by blocking α-amylase [49]. Meanwhile, *S. parviflora* displays antibacterial, anti-fungal, and antiproliferative effects [153].

## 3. Materials and Methods

**Figure 4:**
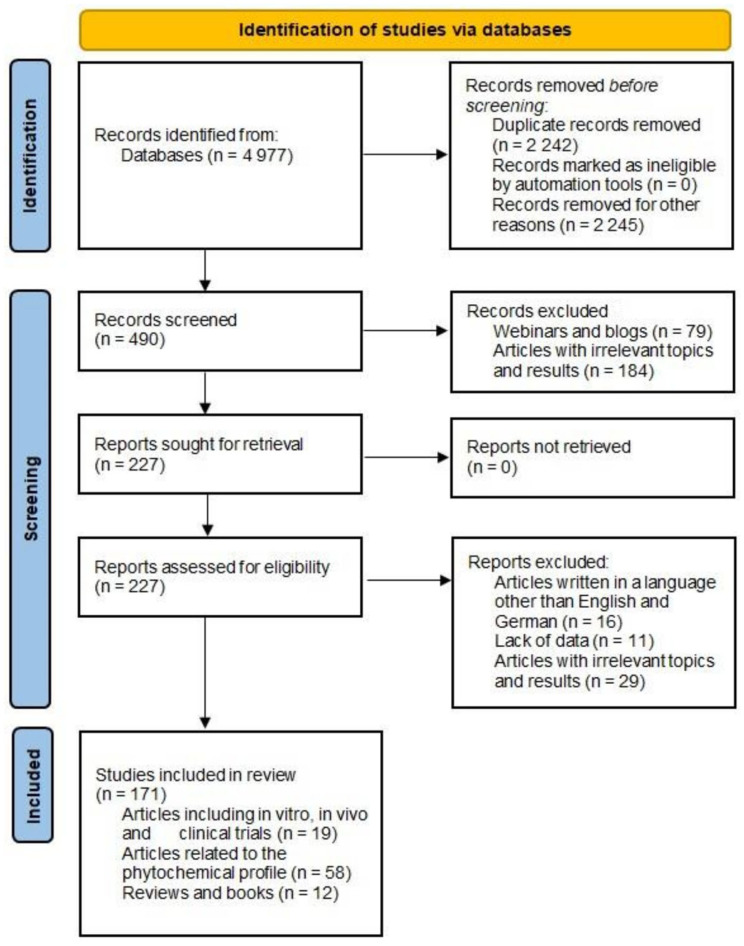
PRISMA 2020 flow diagram [172].

The research methodology comprised a thorough screening process to identify studies focused on the presence and characterization of bioactive phytochemicals in various *Stachys* species, their traditional applications and pharmacological properties. The search strategy employed specific keywords such as: “bioactive compounds”, “Stachys”, “Lamiaceae”, “Stachys extract”, “Stachys essential oil”, “traditional uses”, “chemical composition”, “therapeutic potential”, “clinical trials”, “in vivo studies”, and “in vitro studies”. The keywords were applied individually or in different combinations. Articles published in languages other than English and those considered irrelevant to the subject of the review were omitted. The search strategy was performed following the Preferred Reporting Items for Systematic Reviews and Meta-Analyses (PRISMA) guidelines, presented in Figure 4. A comprehensive search was conducted across the databases Science Direct, PubMed, and Scopus, with no time limit applied during the search process.

## 4. Conclusions

The genus *Stachys* is a diverse and widely distributed group of plants in the Lamiaceae family that is recognized for its significant medical benefits. The plants belonging to this genus have a rich phytochemical profile, including flavonoids, phenolic acids, iridoids, and EOs, which contribute to their great therapeutic potential. Although the genus *Stachys* has long been used to treat a variety of health issues in the traditional medicine of many countries worldwide, there are limited studies that have proven its benefits on health. Only *S. lavandulifolia* has been investigated in clinical trials, and the number of in vivo and in vitro studies on the genus *Stachys* remains limited.

Certain *Stachys* species possess distinct chemical profiles that highlight their potential use in medicine, dietary supplements, and cosmetics. Furthermore, several species such as *S. affinis* and *S. palustris* L. are highly valued for their nutritional qualities.

The broad spectrum of volatile and non-volatile compounds present in *Stachys* species demonstrates their chemical diversity. Many of these substances have shown great therapeutic potential, including antidiabetic, anticancer, anti-inflammation, antibacterial, analgesic, and antioxidant properties.

However, further detailed investigations are required to fully understand and confirm the effectiveness and safety of *Stachys* species for application in medicine.

## Figures and Tables

**Table 1:** Non-volatile constituents of *Stachys* species.

No.	Compound Name	Species	Part	Ref.
Flavonoids and flavonol glycosides				
1	Anisopholin A	*Stachys lanata* Crantz. *	Aerial parts	[42]
2	Annuoside	*Stachys annua* L.	Aerial parts	[43]
3	Apigenin	*Stachys aegyptiaca* Pers.	Leaves and stems	[44,45]
		*Stachys ocymastrum* L. Briq.	Aerial parts	[46]
		*Stachys iva* Griseb. *	Aerial parts	[47]
		*Stachys byzantina* K. Koch	Aerial parts	[48]
		*Stachys cretica* L. subsp. *smyrnaea* Rech Fil.	Aerial parts	[49]
		*Stachys tmolea* Boiss.	Aerial parts	[50]
		*Stachys swainsonii* Benth. subsp. *swainsonii*	Aerial parts	[51]
		*Stachys ionica* Halácsy	Aerial parts	[52]
		*Stachys lavandulifolia* Vahl.	Aerial parts	[53]
4	Calycopterin	*Stachys aegyptiaca* Pers.	Aerial parts	[44,54]
		*Stachys candida* Bory & Chaubard	Aerial parts	[55]
		*Stachys chrysantha* Boiss. & Heldr.	Aerial parts	[56]
5	Casticin	*Stachys ionica* Halácsy	Aerial parts	[52]
6	Chrysoeriol	*Stachys aegyptiaca* Pers.	Leaves and stems	[44,45]
		*Stachys candida* Bory & Chaubard	Aerial parts	[55,56]
		*Stachys chrysantha* Boiss. & Heldr.	Aerial parts	[56]
		*Stachys swainsonii* Benth. subsp. *swainsonii*	Aerial parts	[51]
		*Stachys lavandulifolia* Vahl.	Aerial parts	[53]
7	Chrysoeriol 7-O-β-D-glucopyranoside	*Stachys aegyptiaca* Pers.	Leaves and stems	[44,45]
		*Stachys candida* Bory & Chaubard	Aerial parts	[55,56]
		*Stachys chrysantha* Boiss. & Heldr.	Aerial parts	[56]
		*Stachys swainsonii* Benth. subsp. *swainsonii*	Aerial parts	[51]
8	Chrysosplenetin	*Stachys aegyptiaca* Pers.	Leaves and stems	[44]
		*Stachys ionica* Halácsy	Aerial parts	[52]
		*Stachys lavandulifolia* Vahl.	Aerial parts	[53]
9	Eriodictyol	*Stachys swainsonii* Benth. subsp. *swainsonii*	Aerial parts	[51]
10	Eupatilin	*Stachys glutinosa* L.	Aerial parts	[57]
11	Eupatorin	*Stachys swainsonii* Benth. subsp. *swainsonii*	Aerial parts	[51]
12	Hesperidin	*Stachys cretica* L. subsp. *smyrnaea* Rech Fil.	Aerial parts	[49]
13	5-Hydroxyauranetin	*Stachys swainsonii* Benth. subsp. *swainsonii*	Aerial parts	[51]
14	Isoscutellarein	*Stachys aegyptiaca Pers.*	Leaves and stems	[44,45]
		*Stachys inflata* Benth.	Epigeal parts	[58]
		*Stachys candida* Bory & Chaubard	Aerial parts	[55,56]
15	Isorhamnetin	*Stachys swainsonii* Benth. subsp. *swainsonii*	Aerial parts	[51]
16	Isostachyflaside	*Stachys spectabilis* Choisy ex DC.	Epigeal parts	[59]
		*Stachys atherocalyx* C. Koch.	Aerial parts	[60]
17	Isostachyspinoside	*Stachys spinosa* L.	Aerial parts	[61]
18	Kaempferol	*Stachys cretica* L. subsp. *smyrnaea* Rech Fil.	Aerial parts	[49]
19	Kumatakenin	*Stachys lavandulifolia* Vahl.	Aerial parts	[53]
20	Luteolin	*Stachys aegyptiaca* Pers.	Leaves and stems	[44,45]
		*Stachys ocymastrum* L. Briq.	Aerial parts	[46]
21	Luteolin-7-O-β-D-glucoside	*Stachys swainsonii* Benth. subsp. *swainsonii*	Aerial parts	[51]
22	Martinoside	*Stachys germanica* L.	Aerial parts and roots	[62]
		*Stachys thracica* Davidov	Aerial parts and roots	[62,63]
		*Stachys plumosa* Griseb.	Aerial parts	[64]
23	Naringenin	*Stachys aegyptiaca* Pers.	Aerial parts	[45]
24	Pachypodol	*Stachys lavandulifolia* Vahl.	Aerial parts	[53]
25	Penduletin	*Stachys swainsonii* Benth. subsp. *swainsonii*	Aerial parts	[51]
		*Stachys lavandulifolia* Vahl.	Aerial parts	[53]
26	Salvigenin	*Stachys ionica* Halácsy	Aerial parts	[52]
27	Sideritoflavone	*Stachys glutinosa* L.	Aerial parts	[57]
28	Scutellarein	*Stachys inflata* Benth.	Epigeal parts	[58]
29	Stachyflaside	*Stachys inflata* Benth.	-	[65]
		*Stachys atherocalyx* C. Koch.	-	[65]
		*Stachys atherocalyx* C. Koch.	-	[65]
30	Spectabiflaside	*Stachys spectabilis* Choisy ex DC.	Epigeal parts	[59]
		*Stachys atherocalyx* C. Koch.	Aerial parts	[60,65,66]
31	Stachysetin	*Stachys lanata* Crantz. *	Aerial parts	[42]
		*Stachys iva* Griseb. *	Aerial parts	[47]
32	Stachyspinoside	*Stachys bombycina* Boiss.	Aerial parts	[67]
		*Stachys spinosa* L.	Aerial parts	[68]
		*Stachys swainsonii* Benth. subsp. *swainsonii*	Aerial parts	[51]
33	Velutin	*Stachys lavandulifolia* Vahl.	Aerial parts	[53]
34	Viscosine	*Stachys lavandulifolia* Vahl.	Aerial parts	[53]
35	Xanthomicrol	*Stachys aegyptiaca* Pers.	Aerial parts	[44,54]
		*Stachys glutinosa* L.	Aerial parts	[57]
		*Stachys candida* Bory & Chaubard	Aerial parts	[55]
		*Stachys chrysantha* Boiss. & Heldr.	Aerial parts	[56]
		*Stachys ionica* Halácsy	Aerial parts	[52]
Phenolic acids				
36	1-Caffeoylquinic acid	*Stachys recta* L.	Aerial parts	[28]
		*Stachys palustris* L.	Epigeal parts	[69]
37	Caffeic acid	*Stachys palustris* L.	Epigeal parts	[69]
38	Chlorogenic acid	*Stachys candida* Bory & Chaubard	Aerial parts	[55]
		*Stachys iva* Griseb. *	Aerial parts	[47]
		*Stachys cretica* L. subsp. *smyrnaea* Rech Fil.	Aerial parts	[49]
		*Stachys lanata* Crantz. *	Roots	[42]
		*Stachys tmolea* Boiss.	Aerial parts	[50]
		*Stachys recta* L.	Aerial parts	[28]
		*Stachys palustris* L.	Epigeal parts	[69]
		*Stachys officinalis* L. Trevis	-	[70]
		*Stachys thracica* Davidov	Aerial parts and roots	[62,63]
39	4-Hydroxybenzoic acid	*Stachys tmolea* Boiss.	Aerial parts	[50]
Phenylethanoid glycosides				
40	Acteoside	*Stachys candida* Bory & Chaubard	Aerial parts	[55]
		*Stachys iva* Griseb. *	Aerial parts	[47]
		*Stachys recta* L.	Aerial parts	[28]
		*Stachys affinis* Bunge	Tubers	[71]
		*Stachys lavandulifolia* Vahl.	Aerial parts	[72,73]
		*Stachys officinalis* L. Trevis *	Aerial parts	[72]
		*Stachys germanica* L.	Aerial parts and roots	[62]
		*Stachys sylvatica* L.	Aerial parts and roots	[62]
		*Stachys plumosa* Griseb.	Aerial parts	[64]
		*Stachys thracica* Davidov	Aerial parts and roots	[62,63]
41	Aeschynanthoside C	*Stachys byzantina* K. Koch	Aerial parts	[48]
42	Betonyoside A	*Stachys officinalis* L. Trevis *	Aerial parts	[72]
43	Betonyoside B	*Stachys officinalis* L. Trevis *	Aerial parts	[72]
44	Betonyoside C	*Stachys officinalis* L. Trevis *	Aerial parts	[72]
45	Betonyoside D	*Stachys officinalis* L. Trevis *	Aerial parts	[72]
46	Betonyoside E	*Stachys recta* L.	Aerial parts	[28]
		*Stachys officinalis* L. Trevis *	Aerial parts	[72]
47	Betonyoside F	*Stachys officinalis* L. Trevis *	Aerial parts	[72]
48	Campneoside I	*Stachys lanata* Crantz. *	Roots	[42]
		*Stachys recta* L.	Aerial parts	[28]
49	Campneoside II	*Stachys lanata* Crantz. *	Roots	[42]
		*Stachys officinalis* L. Trevis*	Aerial parts	[72]
50	Darendoside B	*Stachys lanata* Crantz. *	Roots	[42]
51	Forsythoside B	*Stachys recta* L.	Aerial parts	[28]
		*Stachys officinalis* L. Trevis *	Aerial parts	[72]
		*Stachys thracica* Davidov	Aerial parts and roots	[62,63]
52	Isoacteoside	*Stachys lanata* Crantz. *	Roots	[42]
		*Stachys recta* L.	Aerial parts	[28]
		*Stachys officinalis* L. Trevis *	Aerial parts	[72]
53	Lavandulifolioside	*Stachys iva* Griseb. *	Aerial parts	[47]
		*Stachys lavandulifolia* Vahl.	Aerial parts	[73,74]
54	Leucosceptoside A	*Stachys iva* Griseb.*	Aerial parts	[47]
		*Stachys affinis* Bunge	Tubers	[71]
		*Stachys lavandulifolia* Vahl.	Aerial parts	[73]
		*Stachys thracica* Davidov	Aerial parts and roots	[62,63]
55	Leucosceptoside B	*Stachys officinalis* L. Trevis *	Aerial parts	[72]
56	Martinoside	*Stachys affinis* Bunge	Tubers	[71]
		*Stachys germanica* L.	Aerial parts and roots	[62]
		*Stachys sylvatica* L.	Aerial parts and roots	[62]
		*Stachys plumosa* Griseb.	Aerial parts	[64]
		*Stachys thracica* Davidov	Aerial parts and roots	[62,63]
57	Parvifloroside A	*Stachys parviflora* Benth.	Whole plant	[75]
58	Parvifloroside B	*Stachys parviflora* Benth.	Whole plant	[75]
59	Rhodioloside	*Stachys lanata* Crantz. *	Roots	[42]
60	Stachysoside A	*Stachys sieboldii* Miq. *	Leaves	[76]
61	Stachysoside B	*Stachys sieboldii* Miq. *	Leaves	[76]
62	Stachysoside C	*Stachys sieboldii* Miq. *	Leaves	[76]
63	Verbascoside	*Stachys byzantina* K. Koch	Aerial parts	[48]
		*Stachys lanata* Crantz. *	Aerial parts	[42]
		*Stachys lavandulifolia* Vahl.	Aerial parts	[73]
		*Stachys thracica* Davidov	Aerial parts and roots	[62,63]
Phenylpropanoid glycosides				
64	Coniferin	*Stachys lanata* Crantz. *	Roots	[42]
65	Syringin	*Stachys lanata* Crantz. *	Roots	[42]
Iridoids				
66	Acetylharpagide	*Stachys glutinosa* L.	Aerial parts	[77]
		*Stachys iva* Griseb. *	Aerial parts	[47]
		*Stachys corsica* Pers.	-	[78]
		*Stachys alpina* L.	Leaves and stem	[79]
		*Stachys germanica* L.	Leaves, inflorescences	[79]
		*Stachys byzantina* K. Koch	Aerial parts	[79]
		*Stachys recta* L.	Aerial parts	[28]
		*Stachys recta* L.	Aerial parts	[79]
		*Stachys palustris* L.	Aerial parts	[79]
		*Stachys sylvatica* L.	Aerial parts	[79]
		*Stachys officinalis* L. Trevis	Aerial parts	[79]
67	Allobetonicoside	*Stachys glutinosa* L.	Aerial parts	[77]
68	Ajugol	*Stachys spinosa* L.	Aerial parts	[68]
69	Ajugoside	*Stachys alpina* L.	Stems, Leaves	[79]
		*Stachys germanica* L.	Leaves, inflorescences	[79]
		*Stachys byzantina* K. Koch	Aerial parts	[79]
		*Stachys recta* L.	Aerial parts	[79]
		*Stachys palustris* L.	Aerial parts	[79]
		*Stachys sylvatica* L.	Aerial parts	[79]
		*Stachys officinalis* L. Trevis	Aerial parts	[79]
70	Aucubin	*Stachys alpina* L.	Stems, Leaves	[79]
		*Stachys germanica* L.	Leaves, inflorescences	[79]
		*Stachys byzantina* K. Koch	Aerial parts	[79]
		*Stachys recta* L.	Aerial parts	[79]
		*Stachys palustris* L.	Aerial parts	[79]
		*Stachys sylvatica* L.	Aerial parts	[79]
		*Stachys officinalis* L. Trevis	Aerial parts	[79]
71	Catalpol	*Stachys alpina* L.	Stems, Leaves	[79]
		*Stachys germanica* L.	Leaves, inflorescences	[79]
		*Stachys byzantina* K. Koch	Aerial parts	[79]
		*Stachys recta* L.	Aerial parts	[79]
		*Stachys palustris* L.	Aerial parts	[79]
		*Stachys sylvatica* L.	Aerial parts	[79]
		*Stachys officinalis* L. Trevis	Aerial parts	[79]
72	Gardoside	*Stachys iva* Griseb. *	Aerial parts	[47]
		*Stachys ionica* Halácsy	Aerial parts	[52]
73	Harpagide	*Stachys glutinosa* L.	Aerial parts	[77]
		*Stachys iva* Griseb. *	Aerial parts	[47]
		*Stachys corsica* Pers.	-	[78]
		*Stachys alpina* L.	Stems, Leaves	[79]
		*Stachys germanica* L.	Leaves, inflorescences	[79]
		*Stachys byzantina* K. Koch	Aerial parts	[79]
		*Stachys recta* L.	Aerial parts	[79]
		*Stachys spinosa* L.	Aerial parts	[68]
		*Stachys affinis* Bunge	Tubers	[71]
		*Stachys palustris* L.	Aerial parts	[79]
		*Stachys sylvatica* L.	Aerial parts	[79]
		*Stachys officinalis* L. Trevis	Aerial parts	[79]
74	Hydroxyipolamiide	*Stachys ocymastrum* L. Briq.	Leaves	[80]
75	Ipolamiide	*Stachys ocymastrum* L. Briq.	Leaves	[80]
76	Ipolamiidoside	*Stachys ocymastrum* L. Briq.	Leaves	[80]
77	Lamiide	*Stachys ocymastrum* L. Briq.	Leaves	[80]
78	Melittoside	*Stachys glutinosa* L.	Aerial parts	[77]
		*Stachys iva* Griseb. *	Aerial parts	[47]
		*Stachys recta* L.	Tubers	[28]
		*Stachys affinis* Bunge	Tubers	[71]
		*Stachys lavandulifolia* Vahl.	Aerial parts	[73]
79	Monomelittoside	*Stachys glutinosa* L.	Aerial parts	[77]
		*Stachys iva* Griseb. *	Aerial parts	[47]
		*Stachys lavandulifolia* Vahl.	Aerial parts	[73]
Diterpenoids				
80	Annuanone	*Stachys annua* L.	Aerial parts	[19]
		*Stachys sylvatica* L.	Aerial parts	[19]
		*Stachys palustris* L.	-	[19]
81	Betonicoside A	*Stachys officinalis* L. Trevis.	Roots	[81]
82	Betonicoside B	*Stachys officinalis* L. Trevis.	Roots	[81]
83	Betonicoside C	*Stachys officinalis* L. Trevis.	Roots	[81]
84	Betonicoside D	*Stachys officinalis* L. Trevis.	Roots	[81]
85	Betonicolide	*Stachys officinalis* L. Trevis.	Roots	[81]
86	Betolide	*Stachys officinalis* L. Trevis.	Roots	[81]
		*Stachys sylvatica* L.	Aerial parts	[19]
		*Stachys germanica* L.	Aerial parts	[19]
		*Stachys plumosa* Griseb. *	-	[19]
87	Roseostachenone	*Stachys glutinosa* L.	Aerial parts	[57]
		*Stachys rosea* (Desf.) Boiss.	-	[19]
88	Stachone	*Stachys annua* L.	Aerial parts	[19]
		*Stachys sylvatica* L.	Aerial parts	[19]
89	Stachysolone	*Stachys aegyptiaca* Pers.	Aerial parts	[54,82]
		*Stachys lavandulifolia* Vahl.	Aerial parts	[83]
		*Stachys annua* L.	Aerial parts	[19]
Triterpenoids				
90	Oleanolic acid	*Stachys spinosa* L.	Aerial parts	[61]
91	Ursolic acid	*Stachys annua* L.	Aerial parts	[43]
Phytosterols and phytoecdysteroids				
92	Deoxyecdysone	*Stachys hissarica* Regel	-	[84]
93	Integristeron A	*Stachys hissarica* Regel	-	[84]
94	Lawsaritol	*Stachys byzantina* K. Koch	Aerial parts	[85]
95	β-Sitosterol	*Stachys byzantina* K. Koch	Aerial parts	[85]
		*Stachys annua* L.	Aerial parts	[43]
		*Stachys spinosa* L.	Aerial parts	[61]
		*Stachys maritima* Gouan	Aerial parts	[86]
96	Polypodin B	*Stachys hissarica* Regel	-	[84]
97	Stigmasterol	*Stachys byzantina* K. Koch	Aerial parts	[39]
		*Stachys spinosa* L.	Aerial parts	[61]
		*Stachys maritima* Gouan	Aerial parts	[86]

* cultivated species.

**Table 2:** Volatile constituents of the EOs of *Stachys* species.

Plant	Plant Collecting Region	Main Volatile Compounds %	Other Volatile Compounds %	Ref.
*Stachys balansae* L.	Turkey	β-Caryophyllene (24.3%), β-Pinene (24.0%), α-Pinene (16.0%), Myrcene (6.7%)	β-Phellandrene (3.0%), α-Terpineol (2.3%), Geranyl acetate (1.0%)	[111]
*Stachys recta* L.	Turkey	Oct-1-en-3-ol (33.8%), Linalol (13.0%), β-Pinene (7.5%)	α-Terpinene (4.0%), α-Pinene (3.9%), β-Caryophyllene (3.7%),	[111]
*Stachys cretica* subsp. *smyrnaea*	Turkey	β-Caryophyllene (51.0%), Germacrene D (32.8%)	α-Humulene (3.1%), β-Elemene (2.1%), δ-Cadinene (2.1%)	[112]
*Stachys viticina* Boiss.	Turkey	β-Caryophyllene (62.3%), Farnesyl acetate (8.9%)	α-Bisabolol (4.4%), Germacrene D (2.9%), Dodecanoic acid (1.5%)	[29]
*Stachys obliqua* Waldst. & Kit.	Turkey	Germacrene D (45.3%), β-Caryophyllene (16.7%), Limonene (8.3%)	α-Cadinene (4.7%), a-Copaene (2.5%), Caryophyllene oxide (2.3%)	[29]
*Stachys sericantha* P.H. Davis	Turkey	Germacrene D (32.4%), β-Caryophyllene (23.2%), α-Cadinene (7.1%), α-Muurolene (5.4%)	Dodecanoic acid (3.8%), α-Copaene (3.1%), δ-Elemene (1.7%)	[29]
*Stachys bayburtensis* R. Bhattacharjee	Turkey	Germacrene D (33.4%), Caryophyllene oxide (6.1%), β-Bourbonene (6.0%), trans-β-Farnesene (5.4%), α-Muurolene (5.4%), Spathulenol (5.2%), Globulol (5.2%)	α-Cadinene (3.4%), Cubenol (2.4%), β-Caryophyllene (2.3%)	[29]
*Stachys huetii* Boiss.	Turkey	Germacrene D (29.8%), β-Caryophyllene (12.4%), β-Bourbonene (9.1%), trans-β-Farnesene (6.1%). Caryophyllene oxide (5.2%), α-Cadinol (5.2%)	Sabinene (3.4%), Spathulenol (2.9%), t-Muurolol (2.9%)	[29]
*Stachys tmolea* Boiss.	Turkey	Germacrene D (22.2%), β-Caryophyllene (19.7%), Valeranone (8.5%), Spathulenol (6.3%)	Bicyclogermacrene (4.8%), α-Cadinene (4.3%), Farnesyl acetate (3.3%)	[29]
*Stachys germanica* L. subsp. *heldreichii* (Boiss.) Hayek	Turkey	Germacrene D (27.1%), β-Caryophyllene (15.7%), Caryophyllene oxide (12.8%)	τ-Muurolol (4.8%), α-Cadinene (3.9%), Spathulenol (3.7%)	[29]
*Stachys germanica* L. subsp. *bithynica* (Boiss.) R.Bhattacharjee	Turkey	Germacrene D (23.2%), β-Caryophyllene (14.8%), α-Copaene (7.7%), Caryophyllene oxide (6.9%), trans-β-Farnesene (6.8%), Spathulenol (5.8%)	α-Cadinene (4.9%), β-Damascenone (2.9%), τ-Muurolol (2.7%), α-Bisabolol (2.7%)	[29]
*Stachys cretica* L. subsp. *bulgarica* Rech.f.	Turkey	Germacrene D (28.2%), β-Caryophyllene (11.8%), Spathulenol (7.4%), Caryophyllene oxide (6.6%), α-Cadinene (5.7%)	trans-β-Farnesene(4.8%), α-Copaene (3.7%), Sabinene (3.1%)	[29]
*Stachys alpina* L.	Croatia	(E)-Nerolidol (22.1%), Nonanal (9.8%), Dodecanal (9.7%), 1-Octen-3-ol (8.7%), Germacrene-D (5.9%)	(E)-2-Hexenal (4.5%), β-Ionone (2.8%), (Z, E)-α-Farnesene (2.2%)	[113]
*Stachys officinalis* L. Trevis	Croatia	Germacrene D (20.1%), (E)-Caryophyllene (14.6%), Caryophyllene oxide (7.9%), α-Humulene (6.7%)	β-Bourbonene (4.6%), β-Elemene (3.8%), α-Selinene (3.6%)	[113]
*Stachys palustris* L.	Croatia	1-Octen-3-ol (24.5%), Caryophyllene oxide (16.2%), (E)-2-Hexenal (16.3%), (E)-Caryophyllene (6.5%)	γ-Muurolene (4.8%), Germacrene D (3.4%), β-Ionone (3.2%)	[113]
*Stachys recta* L. subsp. *recta*	Croatia	β-Ionone (9.2%), Dodecanoic acid (7.0%), δ-Cadinene (6.9%), (E)-Caryophyllene (5.4%)	Germacrene D (3.5%), (E)-Nerolidol (2.1%), Myrcene (1.8%)	[113]
*Stachys salvifolia* Ten.	Croatia	Germacrene D (22.3%), β-Elemene (9.4%), Valeranone (5.3%)	β-Phellandrene (4.9%), (E)-β-Farnesene (4.3%), Bicyclogermacrene (3.1%)	[113]
*Stachys sylvatica* L.	Croatia	α-Pinene (21.4%), Germacrene D (13.6%), β-Pinene (12.3%), (E)-Caryophyllene (9.9%)	δ-Cadinene (4.3%), Caryophyllene oxide (3.3%), Myrcene (2.2%)	[113]
*Stachys germanica* ssp. *heldrei-* *chii* (Boiss) Hayek	Former Yugoslavian Re- public of Macedonia	(E)-Nerolidol (13.5%), Caryophyllene oxide (13.4%), Germacrene D (8.1%), Camphor (5.7%), (Z)-Nuciferyl isobutyrate (5.5%), β-Caryophyllene (5.1%)	Valeranone (4.8%), γ-Cadinene (3.4%), δ-Cadinene (3.3%)	[114]
*Stachys iva* Griseb.	Former Yugoslavian Republic of Macedonia	(Z)-Nuciferyl isobutyrate (14.0%), δ-Cadinene (10.0%), β-Caryophyllene (9.3%), Valeranone (8.7%), α-Copaene (8.4%), Spathulenol (8.1%), Bicyclogermacrene (7.4%), Caryophyllene oxide (5.4%)	Bicycloelemene (4.2%), τ-Cadinol (3.4%), β-Pinene (2.3%)	[114]
*Stachys plumosa* Griseb.	Serbia	ar-Abietatriene (45.5%), Pinocarvone (9.0%), cis-Verbenol (8.2%), Caryophyllene oxide (6.5%)	β-Pinene (4.2%), Myrtenal (2.8%), a-Pinene (2.1%)	[114]
*Stachys scardica* Griseb.	Serbia	γ-Muurolene (13.1%), β-Caryophyllene (10.0%), δ-Cadinene (9.3%), Caryophyllene oxide (8.2%)	α-Pinene (4.6%), β-Bourbonene (4.6%), γ-Cadinene(4.1%), α-Copaene (3.5%)	[114]
*Stachys officinalis* L. Trevis	Serbia	Germacrene D (19.9%), β-Caryophyllene (14.1%), α-Humulene (7.5%)	δ-Cadinene (4.0%), β-Bourbonene (3.8%), α-Selinene (3.4%)	[115]
*Stachys spruneri* Boiss.	Greece	(+)-Limonene (17.3%), α-Selinene (11.0%), (+)-(E)-Caryophyllene (8.7%), (+)-α-Pinene (8.0%)	δ-Cadinene (4.5%), Sabinene (4.1%), α-Copaene (3.9%)	[116]
*Stachys ionica* Halácsy	Greece	(E)-Neolidol (14.9%), α-Cadinol (13.1%), Spathulenol (7.6%), Torreyol (7.6%), δ-Cadinene (6.2%) Sclarene (6.0%)	Elemol (4.6%), τ-Cadinol (4.6%), Valencene (2.4%), γ-Cadinene (2.3%)	[116]
*Stachys spinosa* L.	Greece	Carvacrol (27.9%), Thymol (4.5%), Caryophyllene oxide (4.4%), Pulegone (4.1%)	Hexahydrofarnesyl acetone (2.8%), Dihydroactinidiolide (2.1%), (Z)-Caryophyllene (1.0%)	[23]
*Stachys balansae* Boiss & Kotschy	Iran	Germacrene D (16.4%), α-Pinene (12.1%), β-Pinene (11.9%), Valeranone (10.4%), Heneicosane (5.9%)	Cembrene (4.7%), Tricosane (3.1%), Bicyclogermacrene (2.5%)	[117]
*Stachys schtschegleevii* Sosn.	Iran	Germacrene D (25.8%), Limonene (8.8%), Valencene (6.1%), α-Pinene (5.6%)	Bicyclogermacrene (4.5%), δ-Cadinene (3.3%), Spathulenol (3.3%), Carvone (2.6%)	[117]
*Stachys byzantina* K. Koch	Iran	Hexahydrofarnesyl acetone (25.7%), Valeranone (17.1%), *n*-Hexadecanoic acid (10.9%), *trans*-Phytol (6.9%), 1-octen-3-ol (6.6%)	α-Bisabolol (3.9%), Caryophyllene oxide (3.1%), (*E*)-β-ionone (1.8%)	[118]
*Stachys germanica* L.	Bulgaria	Camphor (52.96%), Geranyl-p-cymene (10.49%), (E)-β-Farnesene (3.97%)	Trans-Chrysanthenyl acetate (2.66%), Bornyl acetate (2.54%), Camphene (2.52%)	[119]
*Stachys palustris* L.	Italy	Caryophyllene oxide (7.8%), Hexahydrofarnesyl acetone (7.4%), (Z)-Phytol (6.4%), Thymol (5.8%), p-Methoxyacetophenone (5.1%)	4-Vinylguaiacol (3.8%), (E)-Caryophyllene (3.6%), β-Ionone (3.3%)	[23]
*Stachys nivea* Labill.	Lebanon	Spathulenol (6.7%), 4-Vinylguaiacol (6.1%), Germacrene D (2.5%)	Thymol (2.2%), Carvacrol (2.0%), (Z)-b-Damascenone (1.5%)	[23]

**Table 3:** *Stachys* species and their traditional therapeutic uses.

*Stachys* Species	Localization	Traditional Therapeutic Uses	Plant Part/Preparation	Ref.
*Stachys affinis* Bunge (=*Stachys sieboldii* Miq.) =Chinese artichoke	China, Japan, and Korea	Food; treatment for infections, respiratory disorders, colds, gastrointestinal issues, dementia; antioxidant, neuroprotective effects	Tubers	[71,136,137,138,139,140]
*Stachys annua* L.	Mediterranean region	Topical use for relieving headaches	Leaf infusion	[32]
*Stachys annua* subsp. *annua*	Italy	Anticatarrhal, tonic effects	Aerial parts	[141]
*Stachys annua* subsp. *annua* var. *lycaonica*	Turkey	Treatment for colds, antipyretic effect	Decoction	[142]
*Stachys balansae Boiss. & Kotschy*	-	Management of cardiac neuroses and hypotonic conditions	Extract	[143]
*Stachys byzantina* K. Koch	Iran	Treatment for infected wounds	Decoction	[144]
	Brazil	Anti-inflammatory properties	Infusion	[135]
*Stachys cretica* L. subsp. *anatolica*	Turkey	Management of respiratory infections and gastrointestinal disorders	Infusion, decoction	[142]
Stachys floridana Schuttl. ex Benth	China	Treatment for diabetes and colon cancer; antiproliferative effect	-	[41,145]
*Stachys germanica* L.	Iran	Dermatological conditions in veterinary medicine	Infusion	[32,146]
*Stachys glutinosa* L.	Mediterranean region	Antispasmoic effect, against chicken louse	-	[32]
*Stachys inflata Benth.*	Iran	Treatment for cold, pain relief; management of hypertension	Decoction	[147]
		Treatment for inflammation, asthma, infection, and rheumatism	Extract of the aerial parts	[148]
*Stachys iberica* Bieb. subsp. *Georgica* Rech.	Turkey	Treatment for cold; antipyretic action	Decoction	[142]
*Stachys iva* Griseb.	Greece	Prevention of the common cold in the winter	-	[47]
*Stachys kurdica* Boiss & Hohen var. *kurdica*	Turkey	Treatment for abdominal discomfort and cold	Decoction	[134]
*Stachys mucronata* Sieb.	Greece	Topically used in massage to relieve neuralgic and rheumatoid arthritis symptoms; cleansing wounds and ulcers, followed by application of fresh leaf poultice for faster healing	Decoction	[149]
		Antidiarrheal action	Infusion of fresh leaves	
		Purgative effect	Infusion of roots	
*Stachys lanata* Crantz.	-	Management of hypotonic conditions and cardiac neuroses	Extract	[143]
*Stachys lavandulifolia* Vahl.	Turkey	Treatment of anxiety disorders	Extract	[5,142]
		Antipyretic, anticough action	Infusion	
	Iran	Pain relief, anti-inflammatory, anxiolytic, and sedative effects	Boiled extract of the aerial parts	[73]
		Treatment for common cold, headache, stomach discomfort, kidney stones	Decoction of aerial parts	[147]
*Stachys obliqua* Waldst. & Kit.	Turkey	Management of symptoms of cough, fever, stomach aches, and common colds	Herb, infusion, decoction	[34]
*Stachys officinalis* Trev.	Italy	Dermatological conditions in veterinary medicine	Oily extract of flowers	[150]
	Serbia, Montenegro, Egypt	Antibacterial properties; against headaches, nervous tension, anxiety, and menopausal symptoms; helps in quitting smoking	Tea from dried leaves	[34]
	-	Cholagogic and carminative agent; help against headaches	Leaves	[151]
	The Mediterranean region	Dermatological conditions in veterinary medicine	-	[32]
*Stachys parviflora* Benth.	-	Management of camps, joint discomfort, convulsions	-	[152,153]
*Stachys pilifera* Benth	Iran	Anti-infective, anti-asthmatic, anti-rheumatic, analgesic effects	Tea from the aerial parts	[154]
		Help against toothaches; reduces edema; expectorant and anti-cough action	Decoction	[147]
*Stachys palustris* L.	-	Desinfectant; treatment of spasms and wounds	-	[39]
	Poland, Scandinavia	Antiseptic and antihemorrhagic effects; Treatment of gout symptoms	-	[151]
*Stachys recta* L.	Italy	Facial washing for relieving headaches	Infusion of leaves	[155]
		Protection against negative influences or spirits	Decoction	[150,155]
*Stachys sylvatica* L.	-	Desinfectant; treatment of spasms and wounds	-	[39]
	Iran	Diuretic, sedative, tonic, antispasmodic, digestive, and anti-inflammatory properties	-	[156]
	Turkey	Help against heart problems	Infusion of aerial parts	[157]
	-	Contraction of the uterus in animals	Extract	[143]
*Stachys* *tibetica* Vatke	India	Antipyretic, antitussive effects; management of various mental conditions	Decoction	[158]
*Stachys* *turcomanica* Trautv.	Iran	Treatment against influenza, bronchitis, toothache, and foot inflammation	Infusion	[146]

**Table 4:** Clinical studies on *Stachys* species.

Study Objective	Study Design	Main Results	Ref.
Evaluation of the effects on oxidative stress of *S. lavandulifolia*	Twenty-six participants consumed 3 g of aqueous extract of *S. lavandulifolia.* Study duration: 14 days.	Increased antioxidant levels in the blood (*p* = 0.001) and reduced lipid peroxidation (*p* = 0.0001) after treatment with *S. lavandulifolia*.	[161]
Examination of the effects of *S. lavandulifolia* on the management of polycystic ovary syndrome	Sixty-six women, aged 15 to 45 years. Thirty-three were treated with 10 mg of medroxyprogesterone acetate. Thirty-three received 5 g of *S. lavandulifolia* in the form of tea. Period of study: 3 months.	Comparable efficacy of *S. lavandulifolia* to medroxyprogesterone acetate in treating polycystic ovary syndrome—*p* < 0.001. Less side effects of *S. lavandulifolia*—*p* = 0.099.	[162]
Assessment of the influence of *S. lavandulifolia* on menstrual pain	Twenty-nine women, aged 18 to 45 years, received either the *S. lavandulifolia* or placebo capsules every 4–6 h. Period of study: 3 months.	Reduction in the intensity of menstrual pain. *p* < 0.05, compared to placebo.	[163]
Examination of the analgesic effect of *S. lavandulifolia* on migraine pain	Fifty patients were divided into two groups. The intervention group received one cup of *S. lavandulifolia* (10 g in 200 cc water) three times a day, in addition to their standard medication. The patients in the control group were administered routine drugs and a placebo. Period of study: 2 months.	Reduced pain levels during *S. lavandulifolia* treatment. *p* < 0.001, compared to control group.	[164]

**Table 5:** In vivo studies on certain *Stachys* species.

Study Objective	Study Design	Main Results	Ref.
Examination of the antinociceptive effects of *S. lavandulifolia* essential oil	Male Swiss mice were pretreated with essential oil of *S. lavandulifolia* (25 or 50 mg/kg, p.o.), α-bisabolol (25 or 50 mg/kg, p.o.), morphine (3 mg/kg, i.p.), or vehicle, before receiving formalin (20 μL, 2%), capsaicin (20 μL, 2.5 μg), or glutamate (20 μL, 25 mM) in the upper lip. The anti-inflammatory effects of the essential oil of *S. lavandulifolia* or α-bisabolol (50 mg/kg) were asessed using the carageenan (2% in 0.2 mL) pleurisy model. Study duration: 40 min.	Significant inhibitory effect on nociceptive orofacial pain with oral treatment with *S. lavandulifolia* essential oil and α-bisabolol. Variations in the *p*-values were noted across different concentrations and conditions, including orofacial nociceptive behavior: *p* < 0.01, *p* < 0.001, *p* < 0.05.	[165]
Examination of the anti-inflammatory effects of *S. lavandulifolia* essential oil	Male Swiss mice were divided into three groups. Before carrageenan-induced pleurisy, the control group received 10 mg/kg indomethacin, and the other animals were administered oral *S. lavandulifolia* essential oil (50 mg/kg) or α-bisobolol (50 mg/kg). Study duration: 4 h.	Reduction In TNF-α and IL-1β levels and remarkable anti-inflammatory effect after treatment with α-bisabolol and essential oil of *S. lavandulifolia. p* < 0.05, *p* < 0.01 versus control groups.	[165]
Assessment of the effect of *S. sieboldii* on blood glucose levels	Mice were subjected to a fifteen-hour fasting prior to the test day, following BCAO. Fasting blood glucose levels exhibited a significant increase after BCAO in comparison to those of the sham group. The mice were administered chorogi extract, ginkgo extract, or water.	Chorogi extract and ginkgo extract effectively suppressed elevation in fasting blood glucose levels after BCAO when compared to the BCAO group with water. *p* < 0.05.	[139]
Examination of the effect of *S. sieboldii* on learning and memory	Mice received chorogi extract, ginkgo extract, or water once daily for five days. The next day, mice underwent BCAO. Study duration: 6 days.	Following BCAO, a notable dose-dependent reduction in response latency was observed. This decrease was mitigated by both the chorogi and the ginkgo extracts compared to the water-treated control group. *p* < 0.05.	[139]
Examination of the effects of *S. sieboldii* on memory	Male rats were assigned to one of four groups, with 10 rats in each group. The control group received vehicle i.p. and p.o. The second group received scopolamine i.p. and vehicle p.o. The positive control group was administered scopolamine i.p. and donepezil at 5 mg/kg p.o. The last group received scopolamine i.p. and an extract of *S. sieboldii* at doses of 250 or 500 mg/kg p.o. Period of study: 33 days.	Pretreatments with an extract of *S. sieboldii* prevented the memory impairment induced by scopolamine. Supplementation with *S. sieboldii* enhanced spatial memory. *p* < 0.05.	[166]
Evaluation of the antinephritic effect of *S. sieboldii*	Male rats were divided into three groups, each consisting of five rats. Two of these groups received a once-daily oral administration of 30 mg/kg acteoside from the aerial part of *S. sieboldii* and 20 mg/kg cyclosporine A, respectively. The control group was administered distilled water. Study duration: 15 days	Acteoside effectively inhibited the accumulation of CD4-, CD8-, and IL-2-receptor-positive cells in the glomeruli of rats afflicted with crescentic-type anti-GBM nephritis. Variations in the *p*-values were observed in the different effects: *p* < 0.01, *p* < 0.05, compared to the control group. *p* < 0.05, *p* < 0.01, compared to the normal rats.	[167]
Evaluation of the effects of *S. sieboldii* on weight gain and dyslipidemia	Male rats were allocated into four groups, each consisting of eight rats. These groups included rats fed either a regular diet, a high-fat and high-cholesterol (HFC) diet, a *HFC diet* supplemented with 3% of *S. sieboldii*, or a HFC diet supplemented with 5% of *S. sieboldii*. Period of study: 6 weeks.	Supplementation with *S. sieboldii* progressively inhibited weight gain in a dose-dependent way—*p* < 0.001. Oral consumption of *S. sieboldii* significantly improved lipid profiles—variations in *p*-values were observed across different parameters in the lipid profile. *p* < 0.01, *p* < 0.001.	[168]
Examination of the antinociceptive effect of *S. glutinosa*	Male mice were treated with either saline at 40 mg/kg or xanthomicrol (from the extract of the aerial parts of *S. glutinosa*) at 80 mg/kg 30 min preceding the administration of saline or morphine at 5 mg/kg. Baseline nociception was measured immediately prior to the administration of saline or xanthomicrol. The effects were assessed at 30, 60, and 120 min intervals post-morphine administration. Study duration: 120 min.	Intraperitoneal application of xanthomicrol extracted from *S. glutinosa* elicited a notable decrease in morphine-induced antinociception in mice. *p* < 0.05.	[57]
Assessment of the wound-healing effect of *S. hissarica*	Rats were treated with an extract of *S. hissarica* administered orally at a repeated dosage of 10 mg/kg. Methyluracil was used as the control substance.	The healing process in rats with incisional lnear wounds was notably accelerated upon treatment with extract from *S. hissarica* compared to the control group. *p* < 0.001.	[84]
Examination of the effects of *S. sylvatica* on polycystic ovary syndrome	Thirty adult female rats were distributed into five groups, each containing six rats: control, polycysic ovary syndrome group (PCOS) (induced by a single i.m. injection of 2 mg estradiol valerate), and three groups treated with hydroalcoholic extract of *S. sylvatica.* Following 60 days of PCOS induction, *S. sylvatica* extract was administered i.p. at doses of 100, 250, and 500 mg/kg. The control group received only olive oil.	The extract of *S. sylavtica* demonstrated potential for ameliorating obesity in the PCOS group. Following treatment with *S. sylvatica* extract at a dosage of 500 mg/kg, a notable increase was observed in all tested hormones. *p* < 0.001, *p* < 0.01.	[156]
Evaluation of the antiangiogenic action of iridoids from *S. ocymastrum*	Zebrafish embryos were utilized. They were exposed to 100 μL of embyo water containing either iridoids isolated from *S. ocymastrum* (2 or 4 μM) or 2 μM 2-methoxyestradiol, which served as a standard antiangiogenic agent. 0.2% *v*/*v* DMSO acted as a vehicle for the treatments, while the control group received solely DMSO. Study duration: 48 h.	Among the compounds isolated from *S. ocymastrum*, β-hydroxyipolamiide and ipolamiide exhibited the most pronounced suppression of blood vessel growth in zebrafish embryos. *p* < 0.05, *p* < 0.01 versus control group.	[80]
Examination of the nephroprotective effect of *S. pilifera*	Adult rats were divided into five groups, each with seven rats. Cisplatin was administered intraperitoneally at a dose of 7 mg/kg to induce toxicity. The hydroalcoholic extract of *S. pilifera* was orally given at a dose of 500 mg/kg. The control group received intraperitoneal pretreatment with normal saline, followed by distilled water. The second group received distilled water for 12 days and a dose of cisplatin on the fifth day. Group three received the extract of *S. pilifera* for 12 days and a single dose of cisplatin on the fifth day. Group four was given a single dose of cisplatin on the fifth day, followed by the extract for seven days. The last group received only the extract. Study duration: 12 days.	Pretreatment and posttreatment with the hydroalcoholic extract of *S. pilifera* significantly decreased BUN and Cr levels but notably increased total protein levels. Variations in the *p*-values were observed in different concentrations: *p* < 0.05, *p* < 0.01, *p* < 0.001.	[154]
Examination of the hepatoprotective effect of *S. pilifera*	Fourty-two adult male rats were divided into six groups, each comprising seven rats. Group one received olive oil and normal saline; group two received CCl4 and normal saline; and groups three, four, and five received CCl4 along with *S. pilifera* extract at doses of 100, 200, and 400 mg/kg/d, respectively. The sixth group solely received *S. pilifera* extract at a dose of 400 mg/kg/d. Period of study: 60 days.	Pretreatment with the ethanolic extract of *S. pilifera* at doses of 200 and 400 mg/kg/f significantly reduced the CCl4-induced elevation in serum levels of ALT, AST, and ALP. *S. pilifera* extract effectively restored the serum levels of TP and ALB to near-normal levels. *p* < 0.01.	[169]
	Adult male rats were divided into four groups: a control group receiving distilled water; a group administering Acetaminophen (APAP) at a dosage of 2 g/kg; a group treated with APAP along with a hydroalcoholic extract of *S. pilifera* at a dose of 500 mg/kg; and a positive control group receiving APAP along with 100 mg/kg of Silymarin. Study duration: 7 days.	Treatment with the ethanolic extract of *S. pilifera* notably reduced AST and ALT. Treatment with the hydroalcoholic extract of *S. pilifera* led to an increase in total thiol content. *p* < 0.05 versus control group and *p* < 0.05 versus APAP group.	[170]

**Table 6:** In vitro studies on certain *Stachys* species.

Study Objective	Study Design	Main Results	Ref.
Assessment of the cytotoxic effect of *S. aegyptiaca* against the human hepatocellular cell line HepG2	Stachaegyptin D, stachyaegyptin E, stachysolon monoacetate, and stachysolon diacetate, all isolated from an extract of *S. aegyptiaca*, underwent evaluation against human hepatocellular carcinoma cell lines (HepG2).	The highest efficacy against HepG2 cells is shown in stachysolon diacetate (IC_50_ = 59.5 μM), which induced a stronger concentration-dependent decrease in cell proliferation compared to a DMSO solvent control.	[171]
Evaluation of the antioxidant activity of *S. cretica* subsp. *smyrnaea*	A metal-chelating activity assay was employed to assess the chelating ability of the ethyl acetate, methanol, and water extracts of *S. cretica* subsp. *smyrnaea*.	The methanol extract of *S. cretica* subsp. *smyrnaea* exhibited the highest chelating activity (4.82 mg EDTAEs/g dry plant) among the tested samples. It also possessed strong total antioxidant activity (7.06 mg GAFs/g dry plant) and demonstrated high radical scavenging capacity in both DPPH and ABTS assays (9.10 and 17.36 mg Tes/g dry plant, respectively). *p* < 0.05.	[49]
Examination of the inhibitory activity of *S. cretica* subsp. *smyrnaea* extracts against enzymes	Ethyl acetate, methanol, and water extracts of *S. cretica* subsp. *smyrnaea* were employed. These extracts were tested against acetylcholinesterase, butyrylcholinesterase, tyrosinase, and α-amylase.	The methanol extract of *S. cretica* subsp. *smyrnaea* displayed moderate inhibitory effects against acetylcholinesterase (343.78 μg GALAEs/g dry plant) and potent antidiabetic activity against α-amylase (61.47 mg ACEs/g dry plant), with the highest inhibitory effect against α-glucosidase (47.84 mg ACEs/g dry plant). The ethyl acetate extract exhibited the most pronounced inhibitory activity against butyrylcholinesterase (167.68 μg GALAEs/g dry plant) and promising activity for tyrosinase inhibition (2.45 mg KAEs/g dry plant). *p* < 0.05.	[49]
Assessment of the antibacterial properties of *S. parviflora*	St. aureus, B. cereus, and St. epidermidis, E. coli, S. typhi, and Ps. aeruginosa were employed as bacterial strains. The antibacterial efficacy of both the extract and essential oil of *S. parviflora* was investigated, with gentamicin serving as the control substance. Negative and positive controls were included.	The antibacterial activity of the extract was modest, but the essential oil demonstrated significant efficacy against the microorganisms tested, noteworthy against B. cereus (MIC = 0.01 μg/mL) and S. aureus (MIC = 0.1 μg/mL), surpassing that of gentamicin.	[153]
Assessment of the anti-fungal activity of *S. parviflora*	The anti-fungal activity of the methanolic extract and essential oil of *S. parviflora* was assessed against C. albicans. Clotrimazole was used as the control substance.	The anti-fungal activity of the essential oil of *S. parviflora* against C. albicans (MFC = 0.28 μg/mL) was found to be more potent than that of clotrimazole (MFC = 0.78 μg/mL). Conversely, the methanolic extract exhibited no anti-fungal activity (MFC > 9.10 mg/mL).	[153]
Evaluation of the antiproliferative activity of *S. parviflora*	The cytotoxicity of the methanolic extract and EO of *S. parviflora* against three human cancer cell lines–human ovarian carcinoma, human colon carcinoma, and mouse melanoma cell lines–was performed. Doxorubicin was used as a positive control.	The methanolic extract showed no cytotoxic activity against any of the cancer cell lines tested (IC_50_ > 100 μg/mL). Conversely, the essential oil of *S. parviflora* exhibited potent cytotoxic effects against three different cell lines (IC_50_ = 16.55 μg/mL, 26.95 μg/mL, 30.95 μg/mL, respectively), demonstrating a concentration-dependent response. *p* < 0.05.	[153]

## Data Availability

Data are contained within the article.
